# Deregressed EBV as the response variable yield more reliable genomic predictions than traditional EBV in pure-bred pigs

**DOI:** 10.1186/1297-9686-43-38

**Published:** 2011-11-09

**Authors:** Tage Ostersen, Ole F Christensen, Mark Henryon, Bjarne Nielsen, Guosheng Su, Per Madsen

**Affiliations:** 1 The Danish Agricultural and Food Council, Pig Research Centre, Breeding and Genetics, Axeltorv 3, DK-1609, Denmark; 2 Aarhus University, Faculty of Agricultural Sciences, Department of Genetics and Biotechnology, Blichers Allé 20, P.O. BOX 50, DK-8830, Tjele, Denmark; 3 School of Animal Biology, University of Western Australia, 35 Stirling Highway, Crawley WA 6009, Australia

## Abstract

**Background:**

Genomic selection can be implemented by a multi-step procedure, which requires a response variable and a statistical method. For pure-bred pigs, it was hypothesised that deregressed estimated breeding values (EBV) with the parent average removed as the response variable generate higher reliabilities of genomic breeding values than EBV, and that the normal, thick-tailed and mixture-distribution models yield similar reliabilities.

**Methods:**

Reliabilities of genomic breeding values were estimated with EBV and deregressed EBV as response variables and under the three statistical methods, genomic BLUP, Bayesian Lasso and MIXTURE. The methods were examined by splitting data into a reference data set of 1375 genotyped animals that were performance tested before October 2008, and 536 genotyped validation animals that were performance tested after October 2008. The traits examined were daily gain and feed conversion ratio.

**Results:**

Using deregressed EBV as the response variable yielded 18 to 39% higher reliabilities of the genomic breeding values than using EBV as the response variable. For daily gain, the increase in reliability due to deregression was significant and approximately 35%, whereas for feed conversion ratio it ranged between 18 and 39% and was significant only when MIXTURE was used. Genomic BLUP, Bayesian Lasso and MIXTURE had similar reliabilities.

**Conclusions:**

Deregressed EBV is the preferred response variable, whereas the choice of statistical method is less critical for pure-bred pigs. The increase of 18 to 39% in reliability is worthwhile, since the reliabilities of the genomic breeding values directly affect the returns from genomic selection.

## Background

Genomic selection in pure-bred pigs can be implemented using a multi-step procedure. Effects of dense genetic markers are estimated using a reference population and these effects are used to predict genomic breeding values (GBV) of selection candidates [1]. Implementing a multi-step procedure relies on two prerequisites: 1) a response variable that summarises the genetic information for reference animals, and 2) a statistical method that associates the response variable to the marker information. The choice of response variable and statistical method may well depend on the data structure. Therefore, the challenge is to find a suitable response variable and statistical method that can handle pure-bred pig data.

Pure-bred pig data often have low and varying reliabilities of estimated breeding values (EBV). Some Duroc pigs in the Danish breeding scheme only have their own records, others only offspring records, while some have both - depending on the trait. Furthermore, only a subset of pigs are genotyped and these pigs tend to be closely related. Therefore, a response variable and a statistical method capable of handling such data are needed.

Among the possible response variables, EBV and deregressed EBV are the most promising to date. Garrick et al. [2] showed, at least in theory, that deregressed EBV with the parent average removed (hereafter referred to as "deregressed EBV") yield more accurate genomic breeding values than EBV for two reasons. First, deregressed EBV as the response variable results in less double-counting compared to EBV, because the proposed deregressed EBV excludes ancestral information. If both an offspring and it's parent are genotyped, the degree of double-counting decreases when using deregressed EBV as the response variable. Second, using EBV as the response variable results in double shrinkage of the genomic breeding values, particularly when the reliabilities of the EBV are low. In dairy cattle, Guo et al. [3] compared the two response variables on simulated data and found that deregressed EBV yielded slightly lower reliabilities. However, their simulated data were characterized by low degrees of double-counting. It appears that most pure-bred pig data, with EBV with low reliabilities and high degrees of double-counting, compared to Guo et al. [3], are more in line with the theoretical expectations. Therefore, this suggests that deregressed EBV as the response variable may yield higher reliabilities of genomic breeding values than EBV.

Three types of statistical models have been widely used in the literature [1,4-14]. These are models that assume the marker effects to be normally distributed, models that assume a thick-tailed distribution of marker effects, and models that assume a mixture of two distributions. The performance of the different models is predominantly affected by the number of QTL, the marker density and the genetic relatedness of the population. However, there is no unambiguous evidence that one model will yield more accurate genomic breeding values for pure-bred pig data for two reasons. First, mixture-distribution models have shown promising results for some traits, in particular traits under weak selection, possibly because these traits can be influenced to a greater extent by a few large QTL [9,15]. Second, low genetic relatedness between reference animals and validation animals appears to favour mixture-distribution models [14], presumably because mixture-distribution models utilize linkage disequilibrium more efficiently than normal and thick tailed distribution models. Probably for the same reason, Hayes et al. [9] concluded that thick-tailed models and models assuming normality were equally good only when data from a single cattle breed were analysed. It appears that for pure-bred pig data, for which there is strong selection on traits of interest and high relatedness between genotyped animals, none of the models would be favoured over the others. Therefore, the models assuming mixture-distribution, thick tailed distribution and normal distribution might yield similar reliabilities of genomic breeding values.

In summary, we reasoned that 1) deregressed EBV as the response variable yields higher reliabilities of the GBV compared to EBV, and that 2) normal, thick-tailed and mixture-distribution models yield similar reliabilities. To test these hypotheses, the three models and the two response variables were assessed for the reliabilities by which they could predict GBV for the two traits, daily gain and feed conversion ratio, in Danish Duroc pigs.

## Methods

### Procedure

Two response variables, EBV and deregressed EBV, and three statistical methods with normal, thick-tailed, and mixture-distributions, were assessed for their reliability to predict GBV for daily gain and feed conversion ratio in Danish Duroc pigs. The reliabilities were computed by splitting the genotyped animals into 1375 reference animals that were performance tested before October 2008 and 536 validation animals that were performance tested after October 2008 (further details are given below).

### Data

The Duroc pigs were part of the genetic evaluation system in Denmark. All data were supplied by the Danish Agriculture and Food Council, Pig Research Centre.

#### Genotyping

A total of 1911 Danish Duroc pigs were genotyped using the Illumina PorcineSNP60 BeadChip (Illumina, San Diego, CA). A total of 26142 SNP markers and each of the animals met the following requirements. Each animal had a call rate greater than 0.95. Each marker was mapped to an autosome, had a minor-allele frequency greater than 0.05, a call-frequency score greater than 0.95, and a heterozygote frequency that did not deviate from Hardy-Weinberg expectations by more than $1∕4\sqrt{pq}$, where *p *and *q *are the allele frequencies at the marker. The $1∕4\sqrt{pq}$ corresponds to a 1/4 standard-deviation unit when assuming a binomial distribution of alleles. For each animal and SNP-genotype combination, the GenCall score was greater than 0.65. Genotypes less than 0.65 were defined as missing. Animals with missing genotypes were allocated the population mean for the missing markers.

#### Performance test and pedigree

Daily gain (g/day) and feed conversion ratio (feed units/kg gain) were observed in the interval 30-100 kg live weight. Recordings were performed in the period 1992 to 2010. The pedigrees of the animals were traced back to 1984, and consisted of 345686 and 52537 animals for daily gain and feed conversion ratio, respectively. Both pedigrees included 373 unknown parents (base animals).

The reference data consisted of available records on October 1st 2008 and included 313068 and 23628 measurements of daily gain and feed conversion ratio, respectively. All of the 1375 genotyped animals in the reference data had their own records for daily gain, whereas only 898 genotyped animals had their own records for feed conversion ratio. There were 680 genotyped animals in the reference data that had more than four offspring records for daily gain and 633 genotyped animals that had more than four offspring records for feed conversion ratio.

The full data consisted of records available until May 2010 and included the records from the reference data and additional 32618 and 3344 measurements of daily gain and feed conversion ratio, respectively. All 536 genotyped validation animals were phenotyped for both daily gain and feed conversion ratio after October 1st, 2008.

### Response variables

The EBV were calculated for daily gain and feed conversion ratio by single-trait animal models, and deregressed EBV were calculated by applying the procedure proposed by Garrick et al. [2].

The single-trait animal models were based on the routine evaluation model, with the regression effect of start weight, fixed effect of herd-week-section, random effect of pen and a random additive genetic effect. The model fitted to daily gain also included a fixed effect for sex and a random effect for birth litter. The variance components were estimated using REML. The heritabilities for daily gain and feed conversion ratio were 0.27 and 0.21. The 1375 reference animals had mean reliabilities for EBV of 0.62 (*sd *= 0.18) and 0.36 (*sd *= 0.12) for daily gain and feed conversion ratio, respectively. The software DMU [16] was used to estimate variances and predict the breeding values. Single-trait animal models were used because preliminary analyses showed that predictions of GBV were not improved with a bivariate model.

The deregression procedure of Garrick et al. [2] adjusts for ancestral information, such that the deregressed EBV only contains their own and the descendant's information on each animal. The deregression also eliminates shrinkage contained in the EBV, and therefore deregressed EBV behave as though they were observations with a heritability equal to the reliability of the deregressed EBV (reliabilities computed as in Garrick et al. [2]). Deregressed EBV have unequal variances and should be used in a weighted analysis. To ensure the quality of deregressed EBV, only animals with a deregressed reliability above 0.05 were included in the analysis. This resulted in 35 animals being removed from the analyses with deregressed EBV for feed conversion ratio.

### Statistical methods to estimate marker effects

The marker effects were estimated by fitting linear, additive models to the response variables. The model was:

(1) Y=μ+Xβ+ε,

where *Y *is a *n *× 1 vector of responses, with *n *being the number of reference animals. The mean is denoted *μ*, which is a scalar. The coefficient matrix for the markers, *X*, assume the values -1, 0 or 1, and has dimension *n *× *p*, where *p *is the number of markers. The vector of marker effects is denoted *β *and has dimension *p *× 1, whereas the vector of residuals, *ε*, has dimension *n *× 1, and is distributed $\varepsilon \sim N\left(0,\sigma_{\varepsilon }^{2}W\right)$, where *W *is a diagonal matrix with elements *w*_1_, ..., *w_n_*.

For the analyses with EBV as the response variable, *w*_1 _= . . . = *w_n _*= 1, whereas for the analyses with deregressed EBV as the response variable, a weighted analysis was performed according to Garrick et al. [2]. The weight for the *i*th animal was $w_{i}=\left(1-h^{2}\right)∕\left[\left(c+\left(1-r_{i}^{2}\right)∕r_{i}^{2}\right)h^{2}\right]$, where *c*, the part of the genetic variance not explained by markers, was assumed to be 0.1, *h*^2 ^was the heritability of the trait, and $r_{i}^{2}$ was the reliability of the deregressed EBV of the *i*th animal.

The statistical models studied differ by the distributional assumptions made on *β*, and by the shortness of presentation they are referred to by the corresponding inferential procedure. A brief description of each method is provided in the following.

#### GBLUP

The model for genomic BLUP (GBLUP) can be described as in equation (1), where the vector of marker effects are distributed $\beta \sim N\left(0,\sigma_{v}^{2}I\right)$. Alternatively, the model could be written as *Y *= *μ *+ *g *+ *ε*, where the genetic effects $g\sim N\left(0,\sigma_{v}^{2}XX^{T}\right)$, i.e. a model with a genomic relationship matrix [7]. In this study, the latter form was used, where *XX^T ^*for computational reasons was replaced by (*X *- *P*)(*X *- *P*)*^T ^*with *P *containing the row means of *X *across animals. Variance components were estimated using REML, and the GBV were BLUP solutions from the mixed model equations. Software DMU [16] was used to fit GBLUP models.

#### Bayesian Lasso

The model for Bayesian Lasso assumes that the marker effects follow a double exponential distribution [12,17], which is a thick-tailed distribution. This is also referred to as a Laplace distribution. Thus, *β *~ *Laplace*(0, *λI*) where *λ *is a scaling parameter. The MCMC software BayZ [18] was used for estimation. A Gibbs sampler was applied and a total of 40000 iterations of sampling was performed, with a burn-in of 10000 iterations.

#### MIXTURE

The model for MIXTURE assumes that the marker effects follow a mixture of two normal distributions with similar characteristics as the mixture model in Meuwissen [19]. The distributional assumption can be described as: $\beta \sim \pi_{0}N\left(0,\sigma_{\beta 0}^{2}I\right)+\pi_{1}N\left(0,\sigma_{\beta 1}^{2}I\right)$, where *π*_0 _= 0.9, *π*_1 _= 0.1, and $\sigma_{\beta 0}^{2}=0.01\cdot \sigma_{\beta 1}^{2}$. The MCMC software BayZ [18] was used for estimation. A Metropolis-Hastings sampler was applied with a total of 40000 iterations of sampling and a burn-in of 10000 iterations.

### Evaluation criterion

The predictive ability of each method was evaluated by the reliability of the GBV on the genotyped validation animals. To avoid use of overlapping information between the reference and validation animals [20], we based the validation on the own phenotype of genotyped validation animals, adjusted for fixed effects and non-genetic random effects. The computation of adjusted phenotypes for genotyped validation animals was based on the full data, and the adjusted phenotypes were the sum of EBV and the estimated residual errors (ĝ+ê). The reliability of the GBV, $r_{\left(GBV\right)}^{2}$, is the squared correlation $r_{\left(GBV,g\right)}^{2}$ between GBV and the vector of genetic effects, *g*, for genotyped validation animals. Using that the vector of residual errors, *e*, for these animals is independent of the GBV, and that g+e≈ĝ+ê, we obtain a formula for the reliability of the GBV

(2) $$\begin{array}{c} r_{\left(GBV\right)}^{2}=r_{\left(GBV,g\right)}^{2}=r_{\left(GBV,g+e\right)}^{2}\cdot \omega \\ \approx r_{\left(GBV,ĝ+ê\right)}^{2}\cdot \omega , \end{array}$$

where  ĝ  is the vector of EBV for validation animals,  ê  is the estimated residual vector, $\omega =\left(\sigma_{e}^{2}+\sigma_{a}^{2}\right)∕\sigma_{a}^{2}$, $\sigma_{e}^{2}$ is the residual variance and $\sigma_{a}^{2}$ is the additive genetic variance of the trait. The formula above for the reliability is similar to other formulas shown in the literature [14,21].

A test was performed to investigate whether the reliabilities of the GBV from the two methods were significantly different from each other within each trait. The test hypothesis was that the reliability of GBV from method A was equal to the reliability of GBV from method B, i.e. $H_{0}:r_{\left(GBV_{A},ĝ+ê\right)}^{2}\cdot \omega =r_{\left(GBV_{B},ĝ+ê\right)}^{2}\cdot \omega$. Since *ω *is simply a constant for each trait, the test hypothesis can be reduced to $H_{0}:r_{\left(GBV_{A},ĝ+ê\right)}=r_{\left(GBV_{B},ĝ+ê\right)}$. However, ĝ+ê appears in both correlations and the two correlations are not independent within each trait. This issue of testing equality of correlated correlations was addressed by the Hotelling-Williams *t*-test [22,23], which was applied to each trait with a confidence level of 5%.

## Results

### Response variables

The response variable, deregressed EBV, resulted in higher reliabilities than EBV for both feed conversion ratio and daily gain (Table 1). For daily gain, the reliabilities were on average 35% higher for deregressed EBV with GBLUP, Bayesian Lasso and MIXTURE. The reliabilities for daily gain increased from approximately 0.25 to 0.34. For feed conversion ratio, the reliabilities were on average 18% higher for deregressed EBV for GBLUP and Bayesian Lasso, which approached significance (p-values between 0.05 and 0.11). Use of deregressed EBV instead of EBV increased reliabilities from approximately 0.16 to 0.19 for GBLUP and Bayesian Lasso. For the method MIXTURE, the deregressed EBV yielded 39% higher reliabilities than EBV, increasing reliabilities from 0.15 to 0.20.

**Table 1:** Reliabilities of GBV, $r_{\left(GBV\right)}^{2}$, based on the three statistical methods and the two response variables for daily gain and feed conversion ratio.

		$r_{\left(GBV\right)}^{2}$
	
	EBV	deregressed EBV
**Daily gain**^Ŧ^		
GBLUP	0.26^*a*^	0.33^*b*^
Bayesian Lasso	0.25^*a*^	0.34^*b*^
MIXTURE	0.25^*a*^	0.34^*b*^
**Feed conversion ratio**^∓^		
GBLUP	0.16^*ab*^	0.19^*ab*^
Bayesian Lasso	0.16^*ab*^	0.19^*ab*^
MIXTURE	0.15^*a*^	0.20^*b*^

^∓ ^Reliabilities within daily gain and feed conversion ratio with different superscripts are significantly different (*P *< 0.05) Reliabilities for daily gain and feed conversion ratio are not comparable

### Statistical methods

The three statistical methods, GBLUP, Bayesian Lasso and MIXTURE did not yield different reliabilities (Table 1). For daily gain, the reliabilities ranged from 0.33 to 0.34 for deregressed EBV and from 0.25 to 0.26 for EBV. For feed conversion ratio, the reliabilities ranged from 0.19 to 0.20 for deregressed EBV and from 0.15 to 0.16 for EBV.

## Discussion

The hypothesis that deregressed EBV yield higher reliabilities than EBV was supported. The increase in reliability of 18 to 39% for Duroc pigs is worthwhile, since the reliabilities of the genomic breeding values directly affect the returns from genomic selection. The hypothesis that the different statistical methods yielded similar reliabilities was also supported. Therefore, we believe that when applying genomic selection within a pure-bred pig population, deregressed EBV is the preferred response variable, while the choice of the statistical method is less critical.

The fact that the increase in reliabilities due to deregression was so large was surprising, since results reported by Guo et al. [3] suggested that EBV is the more suitable response variable. There are three possible reasons. In our study less information was available for the reference animals compared to Guo et al. [3], which implies that the expected double shrinkage was increased when using EBV as the response variable. The amount of information was more heterogeneous among the reference animals, and this favours deregressed EBV. The heterogeneity in amount of information was larger for daily gain than for feed conversion ratio, which also explains why the effect of deregression was larger for this trait. The combination of less information per reference animal and several parent-offspring relationships within the reference population increases double-counting further. If a genotyped parent has only one offspring, and this offspring is genotyped, the degree of double-counting is much larger than if both the parent and the offspring have many ungenotyped offspring. So, the combination of sire-son relationships with a low and heterogeneous amount of information in the reference data seems to favour deregressed EBV over EBV.

There are two reasons why the three statistical methods performed equally well. First, the traits have been subject to strong selection, which suggests that MIXTURE would not have an advantage over the other methods [9,15]. Second, we considered only one breed, in which the genotyped animals were closely related, implying that Bayesian Lasso and MIXTURE, which utilize linkage disequilibrium more efficiently, have no advantage over GBLUP [9,14]. Thus, the small difference between the statistical methods is caused by the pure-bred pig data with its high relatedness between genotyped animals and traits that have been subject to strong selection.

The results from this study may only partly apply to other species, traits and data structures. The positive effect of using deregressed EBV as the response variable will depend on the degree of double-counting, and the amount and heterogeneity of information for genotyped animals. However, since the deregressed EBV proposed by Garrick et al. [2] are theoretically more appropriate than EBV, we believe that deregressed EBV will be advantageous in most circumstances. In contrast, the conclusion about equal performances of the statistical methods is not generic. For traits controlled by large QTL, data from multiple breeds or distantly related genotyped animals, or when using a denser marker panel, MIXTURE and Bayesian Lasso could outperform GBLUP. The reason for this is that MIXTURE and Bayesian Lasso utilize linkage disequilibrium more efficiently. We believe that the positive effect of using deregressed EBV applies to most situations, whereas choosing the best statistical method is more sensitive to the particular situation.

The applied multi-step method has the benefit of being readily applicable in most breeding schemes. EBV with reliabilities are easily predicted, and can then be used to calculate deregressed EBV [2]. The deregression procedure itself is very simple and only requires little computing power. The association of marker effects with the response variable by GBLUP is relatively simple, since GBLUP is implemented in common breeding value estimation software. Bayesian Lasso and MIXTURE are available in various MCMC software, although particularly MIXTURE is sensitive to the prior information given, which makes the use of these methods less appealing. A final step, which has not been examined in the present study, would be to combine the genomic breeding values with the traditional pedigree information [24,25].

## Competing interests

The authors declare that they have no competing interests.

## Authors' contributions

TO carried out most calculations and drafted the main parts of the manuscript, OFC created the EBVs from the animal model, made the GBLUP predictions, wrote significant parts of the manuscript and guided on statistical issues. MH guided in the structure of the manuscript, constructed the SNP data and wrote parts of the manuscript. BN, GS and PM helped conceive of the study and guided in the statistical analyses. All authors read and approved the final manuscript.
